# Editorial: Insights in pediatric ophthalmology and strabismus: 2022

**DOI:** 10.3389/fopht.2024.1382073

**Published:** 2024-05-15

**Authors:** Dominique Bremond-Gignac

**Affiliations:** ^1^ Ophthalmology Department, Necker-Enfants Malades University Hospital, AP-HP, Paris Cité University, Paris, France; ^2^ INSERM, UMRS1138, Team 17, From Physiopathology of Ocular Diseases to Clinical Development, Sorbonne Paris Cité University, Centre de Recherche des Cordeliers, Paris, France

**Keywords:** gene therapy, retina, children, strabismus, nystagmus, cornea, pediatric

Children are not just small adults. There are certainly specific qualities in the field of pediatric ophthalmology, but they should be conveyed with a strong acknowledgment of the similarities between adults and children. Over the past two decades, we have observed amazing developments in the diagnosis, ocular imaging, and treatment of childhood ocular diseases. The challenges of child visual care pose significant issues across various fields. We are pleased to present a comprehensive collection of original research and review articles in the Research Topic “*Insights in pediatric ophthalmology and strabismus: 2022*”, which discusses the mechanisms of pediatric ocular diseases and innovation in eye diseases. In everyday practical life, pandemic myopia has increased significantly—up to 90% of young adults in some Asian countries—and may affect up to half of the population by 2050 (1). This has raised the issue of the management of ocular complications and loss of quality of life of the children. In children, screening for myopia is essential to detect the onset of eye elongation. Photoscreeners can be highly beneficial for detecting myopia in preschool children, as demonstrated in the study by (Hunter et al.). However, classical refraction under cycloplegia is still required (2). Naturally, environmental factors are one of the key factors of the myopia pandemics, and parents will be advised to educate their children. Furthermore, recent innovative therapies, such as defocus lenses, defocus contact lenses, orthokeratology, and low-dose atropine, now provide efficient treatment for evolutive myopia. Ocular motility anomalies like nystagmus in children can be either primary or secondary and affect head position, creating torticollis. The Anderson Kestenbaum surgical technique, which aims to reduce abnormal head position, is evaluated at 2 years, with satisfactory results, in a study by (Kuziel et al.). Retinopathy of prematurity (ROP) is also a prevalent severe condition due to an increasing number of premature children (3). In the study of Ajjarapu et al., infants were evaluated after treatment for ROP with laser photocoagulation. Of 183 premature babies that underwent ROP laser treatment, the authors found 4,4% of them had late-onset anterior segment complications. These complications affect both the vision and quality of life (QOL) of the babies and may require additional procedures. Complications should also be identified for early detection and to adjust the anterior segment treatment. Congenital cataracts have diverse etiologies (4), and the various embryology mechanisms remain complex. The article by Minogue et al. demonstrates the formation of calcium-containing deposits is an essential event in the development of congenital cataracts. Genetic pediatric eye disease frequently leads to severe visual impairment or even blindness (4). Individualization of genetic ocular diseases allows for better comprehension. Two articles address genetic issues in ophthalmology; one (Martinez Sanchez and Whitman) focused on genetic transmission in strabismus. In the pediatric population, strabismus affects approximately 2%–4% of children (5). The article of reference (5) reviews the evidence for a genetic contribution to strabismus and the recent advances in the most common forms of strabismus, such as comitant strabismus. In strabismus, exome sequencing has not revealed definitive causative variants. However, in conclusion, CNVs (Copy Number Variants) may alter gene regulation by disrupting the proximity of enhancers and silencers to promoters, the SNPs (Single Nucleotide Polymorphism) identified by GWAS affect the regulation of nearby genes, and the environmental risk factors affect epigenetic methylation. The second genetic article focused on the FRMD7 gene (Liu et al.) and identified a novel heterozygous missense mutation FRMD7 gene in a Chinese family. The case report presented an idiopathic congenital nystagmus (ICN) without foveal hypoplasia. So, in this case of FRMD7 X-Linked mutation, ICN might be caused by a developmental defect in the brain’s ocular motor regions that control fixation. As only one family is described, further studies are needed.

The six articles in this 2024 Research Topic: *Insights in pediatric ophthalmology and strabismus: 2022* provide a wide discussion of innovation in visual screening, strabismus techniques, congenital cataracts development, retinopathy of prematurity treatment and evolution, and the genetics of ocular movements including strabismus and idiopathic congenital nystagmus. The specificity of pediatric ophthalmology and children’s care is crucial to providing improved management of ocular diseases in children. A growing number of innovative therapies are the object of further clinical trials. This broad approach will bring advanced data and information to ophthalmologists, geneticists, orthoptists rare eye disease associations, and basic researchers.

## Author contributions

DB: Conceptualization, Data curation, Formal analysis, Funding acquisition, Investigation, Methodology, Project administration, Resources, Software, Supervision, Validation, Visualization, Writing – original draft, Writing – review & editing.
